# Marked improvement in HbA_1c_ following commencement of flash glucose monitoring in people with type 1 diabetes

**DOI:** 10.1007/s00125-019-4894-1

**Published:** 2019-06-09

**Authors:** Victoria Tyndall, Roland H. Stimson, Nicola N. Zammitt, Stuart A. Ritchie, John A. McKnight, Anna R. Dover, Fraser W. Gibb

**Affiliations:** 1 Edinburgh Centre for Endocrinology and Diabetes, Western General Hospital, Edinburgh, UK; 2 Edinburgh Centre for Endocrinology and Diabetes, Royal Infirmary of Edinburgh, Little France Crescent, Edinburgh, UK; 3 Centre for Cardiovascular Science, University of Edinburgh, Edinburgh, EH14 4TJ UK

**Keywords:** Clinical Diabetes, Clinical diabetes, Continuous glucose monitoring, Devices, DKA, HbA_1c_, Human, Hypoglycaemia, Psychological aspects

## Abstract

**Aims/hypothesis:**

Minimal evidence supports the efficacy of flash monitoring in lowering HbA_1c_. We sought to assess the impact of introducing flash monitoring in our centre.

**Methods:**

We undertook a prospective observational study to assess change in HbA_1c_ in 900 individuals with type 1 diabetes following flash monitoring (comparator group of 518 with no flash monitoring). Secondary outcomes included changes in hypoglycaemia, quality of life, flash monitoring data and hospital admissions.

**Results:**

Those with baseline HbA_1c_ ≥58 mmol/mol (7.5%) achieved a median −7 mmol/mol (interquartile range [IQR] −13 to −1) (0.6% [−1.2 to −0.1]%) change in HbA_1c_ (*p* < 0.001). The percentage achieving HbA_1c_ <58 mmol/mol rose from 34.2% to 50.9% (*p* < 0.001). Median follow-up was 245 days (IQR 182 to 330). Individuals not using flash monitoring experienced no change in HbA_1c_ across a similar timescale (*p* = 0.508). Higher HbA_1c_ (*p* < 0.001), younger age at diagnosis (*p* = 0.003) and lower social deprivation (*p* = 0.024) were independently associated with an HbA_1c_ fall of ≥5 mmol/mol (0.5%). More symptomatic (OR 1.9, *p* < 0.001) and asymptomatic (OR 1.4, *p* < 0.001) hypoglycaemia was reported after flash monitoring. Following flash monitoring, regimen-related and emotional components of the diabetes distress scale improved although the proportion with elevated anxiety (OR 1.2, *p* = 0.028) and depression (OR 2.0, *p* < 0.001) scores increased. Blood glucose test strip use fell from 3.8 to 0.6 per day (*p* < 0.001). Diabetic ketoacidosis admissions fell significantly following flash monitoring (*p* = 0.043).

**Conclusions/interpretation:**

Flash monitoring is associated with significant improvements in HbA_1c_ and fewer diabetic ketoacidosis admissions. Higher rates of hypoglycaemia may relate to greater recognition of hitherto unrecognised events. Impact upon quality of life parameters was mixed but overall treatment satisfaction was overwhelmingly positive.

**Electronic supplementary material:**

The online version of this article (10.1007/s00125-019-4894-1) contains peer-reviewed but unedited supplementary material, which is available to authorised users.

## Introduction

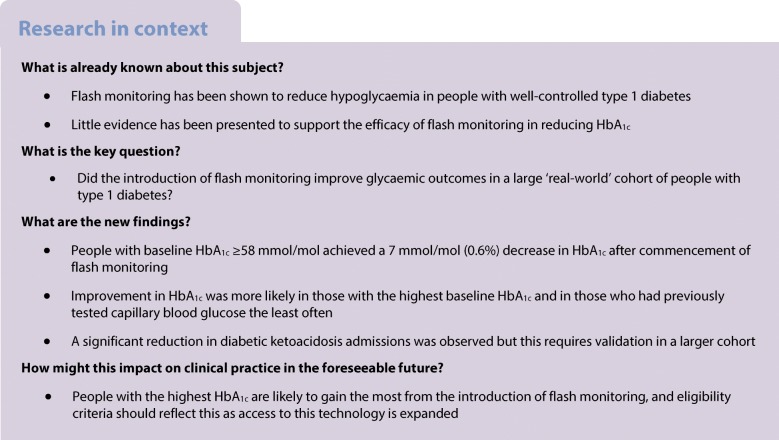



Flash glucose monitoring provides users with an interstitial glucose value upon scanning a glucose sensor with a reader device. It is similar to conventional continuous glucose monitoring (CGM) in providing a 24 h glucose trace and trend arrows to help predict rate of change of glucose. However, unlike CGM, flash monitoring does not provide alarm functions and, in contrast to most current CGM, does not require calibration with blood glucose measurements [1]. Flash monitoring was introduced in the UK in 2015, although prior to November 2017 all use was limited to individuals who self-funded the purchase of glucose sensors. Until recently, therefore, flash monitor use was typically limited to more affluent individuals, with lower than average HbA_1c_ [2]. Most evidence for the effectiveness of flash monitoring in lowering HbA_1c_ comes from small uncontrolled studies [3] and the only large randomised controlled study in type 1 diabetes was limited to people with baseline HbA_1c_ ≤58 mmol/mol (7.5%). This study demonstrated that flash monitoring reduced hypoglycaemia without deterioration in HbA_1c_ [4]. There is, therefore, a paucity of evidence assessing the effectiveness of flash monitoring in a representative population of people with type 1 diabetes. We present the largest prospective evaluation of the impact of flash monitoring in people with type 1 diabetes, with respect to change in HbA_1c_, hypoglycaemia, psychological symptoms, quality of life, flash monitoring data and hospital admissions.

## Methods

### Study design and participants

We conducted a prospective observational study of the first 900 patients commenced on National Health Service (NHS)-funded flash monitoring (Freestyle Libre, Abbott, Witney, UK) in two University hospital clinics (Royal Infirmary of Edinburgh [RIE] and Western General Hospital) during February and March 2018. Prior to February 2018, flash monitor use was limited to those able to self-fund the purchase of sensors. From February 2018 onwards, people with type 1 diabetes were eligible for NHS-funded flash monitor use, conditional upon fulfilling all Scottish Diabetes Group criteria, namely that they: (1) were using intensive insulin therapy; (2) agreed to attend a flash monitoring education session; (3) agreed to scan glucose levels at least six times per day; (4) agreed to share glucose data with their clinic; and (5) had attended a diabetes structured education programme or demonstrated equivalent diabetes self-management knowledge. In February 2018, all people with type 1 diabetes attending our clinics (*n* = 2910) were sent a letter detailing these criteria and, if eligible, how to obtain NHS-funded sensors. All individuals who commenced NHS-funded flash monitoring attended a 1 h education session [5] and completed a form providing the start date and extent of any previous self-funded flash monitor use.

An additional cohort of all individuals with type 1 diabetes attending RIE clinics (where complete HbA_1c_ data were available for each of the past 5 years) was also created (*n* = 1351), for the purpose of tracking longitudinal changes in HbA_1c_, in relation to exposure to flash monitoring. This included a large comparator population with no prior or current flash monitoring exposure (*n* = 518) and also all RIE flash monitor users from the main cohort (described above) where 5 continuous years of HbA_1c_ data were available. This study was entirely observational (with no deviation from standard clinical care) and ethics approval was not required.

### Outcomes

The primary outcome was change in HbA_1c_, defined as the difference between HbA_1c_ prior to commencement of any flash monitoring and the next available value after the flash monitoring education session. We also report the proportion of individuals achieving the Scottish HbA_1c_ target (<58 mmol/mol [7.5%]) and UK National Institute for Health and Care Excellence (NICE) target (≤48 mmol/mol [6.5%]) [6]. We obtained hospital admission and emergency department attendance data for the 6 months following NHS-funded flash monitor use and the corresponding 6 month period in the preceding 2 years. National prescribing database data were obtained for collected prescriptions for glucose test strips and sensors, over the same timescale described above. HbA_1c_, admission and prescribing data are presented for the entire cohort of flash monitor users from both participating hospitals (*n* = 900). Scottish Index of Multiple Deprivation 2016 (SIMD) rank and quintile were determined [7]. The structured education programme offered in our centre is Dose Adjustment for Normal Eating (DAFNE) [8] and previous participation was discerned from our national clinic database system, SCI-Diabetes (https://www.sci-diabetes.scot.nhs.uk). Mode of insulin delivery (multiple daily injection [MDI] or continuous subcutaneous insulin infusion [CSII]) was also obtained from SCI-Diabetes.

Additional data were collected in the subgroup of flash monitor users attending the RIE (*n* = 589): these included change in BMI, clinic questionnaire data, online questionnaire data and flash monitoring data. All individuals attending RIE diabetes clinics are asked to complete a form at each attendance (electronic supplementary material [ESM] Questionnaire 1) which includes hypoglycaemia questions (including Gold score and a modification of the Clarke assessment [9]), frequency of self-monitoring of blood glucose (SMBG), timing of bolus insulin and the Hospital Anxiety and Depression Scale (HADS) [10]. We report changes in hypoglycaemia and HADS score in those where paired pre- and post-flash monitoring questionnaires were available. In addition, all individuals attending RIE flash monitoring education events were sent an online questionnaire invitation 1 month after attendance (ESM Questionnaire 2). This included questions on satisfaction with flash monitoring and a modified version of the diabetes distress scale (DDS) [11]. Flash glucose data was obtained from the ‘LibreView’ portal (Freestyle Libre).

### Statistical analysis

Data were largely non-normally distributed (as determined by Shapiro–Wilk test) and are presented as median and interquartile range (IQR). Paired data were analysed by Wilcoxon signed-rank test and unpaired data by Mann–Whitney *U* test. Comparisons across multiple groups were analysed by Kruskal–Wallis test. Categorical data were analysed by χ^2^ or by McNemar test, when comparing paired repeated measurements. Logistic regression analysis was performed to identify predictors of HbA_1c_ response. One-proportion *Z* test was used to analyse change in modified DDS score responses. Correlations were analysed using Spearman’s rank correlation. A mixed effects model was used to assess the interaction between time (2014–2018) and exposure to flash monitoring on log-transformed HbA_1c_, and paired Student’s *t* test was used to analyse difference in log-transformed HbA_1c_ between 2016 and 2018 for each flash monitoring exposure group. Significance was accepted at *p* < 0.05. All analyses were performed using RStudio version 1.0.153 (https://www.rstudio.com).

## Results

### Baseline characteristics

Baseline characteristics are presented in ESM Table 1. Of the 354 (39.3%) individuals who had a history of previous flash monitoring self-funding, 64.0% reported greater than 50% use prior to NHS funding. Flash monitor use was commenced by 7.4% of self-funders in 2015, 29.9% in 2016, 56.6% in 2017 and 6.0% in 2018.

### Change in HbA_1c_ and BMI following flash monitor use

**Fig. 1:**
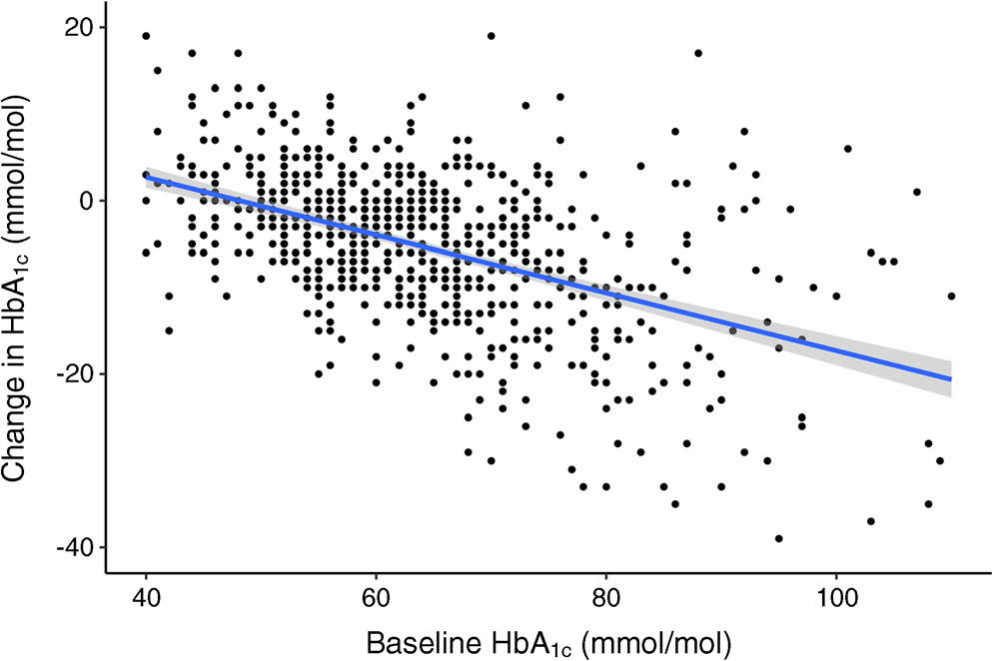
Relationship between baseline HbA_1c_ and subsequent change in HbA_1c_ following flash monitoring. The grey shading indicates the 95% CI for the regression line. Spearman’s *r* −0.479, *p*<0.001

**Fig. 2:**
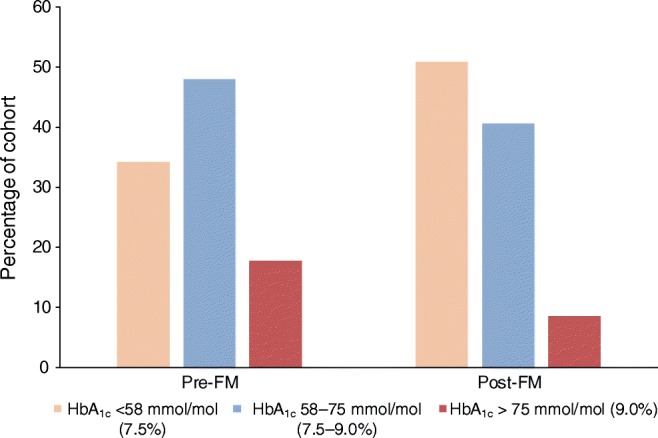
Change in HbA_1c_ category pre- and post-flash monitor use; *p*<0.001, McNemar test for change, in both the <58 mmol/mol (7.5%) and the >75 mmol/mol (9.0%) categories. FM, flash monitoring

The median change in HbA_1c_, between the last value prior to flash monitor use and the most recent value, was −4 mmol/mol (IQR −10 to 0, *p* < 0.001) (−0.4% [−0.9 to 0]). In individuals with baseline HbA_1c_ >75 mmol/mol (9.0%), the median change was −14 mmol/mol (IQR −22 to −7, *p* < 0.001) (−1.3% [−2.0 to −0.6]). In those with baseline HbA_1c_ 58–75 mmol/mol (7.5–9.0%), the median change was −5 mmol/mol (IQR −10 to −1, *p* < 0.001) (−0.5% [−0.9 to 0.1]). In those with HbA_1c_ <58 mmol/mol (7.5%) at baseline, the median change was −1 mmol/mol (IQR −5 to 3, *p* = 0.06) (−0.1% [−0.5 to 0.3]). In total, those with a starting HbA_1c_ that did not meet our national target (<58 mmol/mol [7.5%]) experienced a median −7 mmol/mol (−0.6%) change in HbA_1c_ (IQR −13 to −1 mmol/mol [−1.2 to −0.1%], *p* < 0.001). The median interval from baseline HbA_1c_ to final HbA_1c_ was 245 days (IQR 182 to 330), with no significant correlation between this interval and change in HbA_1c_ (*r* 0.044, *p* = 0.244) (ESM Fig. 1). Baseline HbA_1c_ and subsequent change in HbA_1c_ was strongly negatively correlated (*r* −0.479, *p* < 0.001) (Fig. 1). Overall there was a 48.8% increase in those achieving an HbA_1c_ <58 mmol/mol (7.5%) and a greater than twofold reduction in those with HbA_1c_ above 75 mmol/mol (9.0%) (*p* < 0.001, Fig. 2). The proportion of individuals achieving the NICE target of ≤48 mmol/mol (6.5%) rose from 10.1% to 18.7% (*p* < 0.001). HbA_1c_ since commencement of flash monitoring was not available in 17.4% (*n* = 157) of individuals in this cohort. The only significant differences in individuals with missing follow-up HbA_1c_ were greater proportion of men (20.4% vs 14.4% of women, *p* = 0.017) and more individuals using MDI (19.3% vs 12.4% CSII users, *p* = 0.016) (full data in ESM Table 2). Compared with the corresponding period the previous year, in the first 6 months following NHS-funded flash monitor use, the median number of prescribed (and collected) glucose test strip items (50 test strips per item) fell from 14 (IQR 7 to 21) to 2 (IQR 0 to 6) (*p* < 0.001). This equates to 3.8 test strips per day falling to 0.6 per day. A sufficient number of sensors were collected by 86.5% of flash monitor users to provide complete coverage over the first 6 months (from February or March 2018). Only 3.4% experienced ≤50% sensor coverage across this same period.

Median BMI increased from 26.0 kg/m^2^ (IQR 23.4 to 29.0) to 26.3 kg/m^2^ (23.5 to 29.8) after commencing flash monitoring (median interval 15 months [IQR 5 to 39], *p* < 0.001). Median BMI increase across a similar interval (2016–2018) in 396 non-flash monitor users was 0.1 kg/m^2^ (IQR −0.8 to 1) (*p* = 0.027 comparing BMI change in flash monitor vs non-flash monitor users).

### Predictors of flash monitor use and HbA_1c_ comparison with non-flash monitor users

Within the RIE type 1 diabetes population, those who had not used flash monitoring were older, more likely to be male, had greater social deprivation and were less likely to have an HbA_1c_ <58 mmol/mol (7.5%) at baseline (Table 1).

**Table 1:** Comparison of demographic and clinical features by exposure to flash monitoring

Variable	Self-fund (*n* = 162)	NHS FM (*n* = 250)	Late NHS FM (*n* = 153)	No FM (*n* = 518)	*P* value
Age	42 (30 to 52)	49 (38 to 60)	47 (30 to 57)	54 (38 to 64)	<0.001
Age at diagnosis	16 (10 to 26)	20 (11 to 32)	18 (11 to 29)	23 (12 to 36)	<0.001
Duration of diabetes	22 (13 to 33)	24 (14 to 35)	22 (13 to 33)	24 (15 to 36)	0.254
Female (%)	51.2	53.7	43.8	42.3	0.021
SIMD 1 (%)	6.2	7.3	7.3	15.4	
SIMD 2 (%)	16.3	20.7	22.0	28.3	
SIMD 3 (%)	13.8	19.1	19.3	15.2	
SIMD 4 (%)	16.3	20.3	18.7	16.6	
SIMD 5 (%)	47.5	32.5	32.7	24.6	<0.001
CSII (%)	38.3	24.0	11.1	6.8	<0.001
HbA_1c_ <58 mmol/mol (7.5%) 2016 (%)	35.2	34.4	30.1	25.1	0.016

Data are median (IQR) or %

Continuous variables are compared across all groups by Kruskal-Wallis test and categorical variables by χ^2^ test

Self-fund: individuals who self-funded purchase of flash monitor (FM) prior to taking up NHS-funded sensors in Feb/Mar 2018. NHS FM: individuals whose first FM use was in Feb/Mar 2018 (i.e. no self-funded use). Late NHS FM: individuals whose first FM use was after Mar 2018 (i.e. no self-funded use). No FM: Individuals with no previous or current FM use

**Fig. 3:**
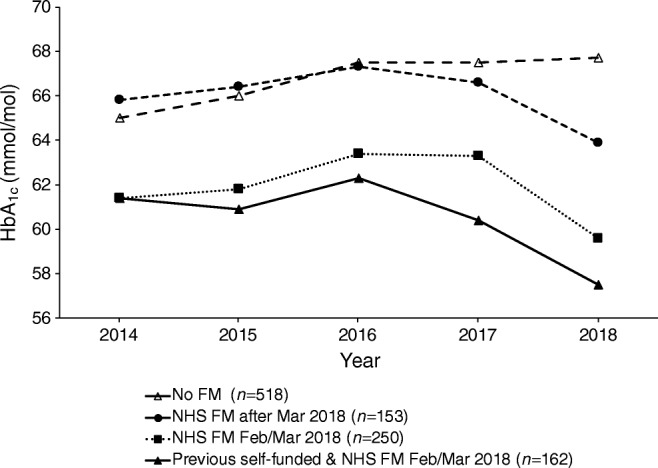
Effect of exposure to flash monitoring on HbA_1c_ trajectory; *p*<0.001 for mixed effects model assessing interaction of time and flash monitoring category on log-transformed HbA_1c_. Wilcoxon signed-rank comparison between 2016 and 2018: No FM, *p*=0.508; NHS FM after Mar 2018, *p*=0.008; NHS FM Feb/Mar 2018, *p*<0.001; Previous self-funded and NHS FM Feb/Mar 2018, *p*<0.001. FM, flash monitoring

Trends in HbA_1c_ were compared in every individual with type 1 diabetes attending the RIE, assessing the influence of flash monitoring exposure. Those who had never used flash monitoring and those who started NHS-funded flash monitoring after the initial February and March 2018 sessions had significantly higher baseline HbA_1c_ than those with a history of self-funded flash monitor use and those commencing flash monitoring for the first time in February and March 2018 (Fig. 3). Between 2016 and 2018 there was a significant fall in HbA_1c_ in all flash monitoring exposed groups but no significant change in those not using flash monitoring (Fig. 3 and ESM Table 3).

### Predictors of fall in HbA_1c_ following flash monitor use

48.1% (361/750) individuals achieved an HbA_1c_ reduction of 5 mmol/mol (0.5%) or greater following commencement of flash monitoring. Univariate analysis identified younger age at diagnosis (18 years [IQR 11 to 29] vs 20 years [12 to 24], *p* = 0.03), higher baseline HbA_1c_ (68 mmol/mol [60 to 78] vs 58 mmol/mol [51 to 65], *p* < 0.001) (8.4% [7.6 to 9.3] vs 7.5% [6.8 to 8.1]) and prior SMBG fewer than four times per day (67.7% vs 45.2%, *p* < 0.001) as predictive of a fall in HbA_1c_ of 5 mmol/mol (0.5%) or greater. Age, diabetes duration, sex, previous self-funding, SIMD quintile, CSII use and DAFNE attendance were not associated with a greater likelihood of achieving a 5 mmol/mol (0.5%) fall in HbA_1c_ with flash monitoring (ESM Table 4). Logistic regression analysis identified higher baseline HbA_1c_ (OR 1.07 [95% CI 1.05, 1.08] per mmol/mol increment [0.1%]; *p* < 0.001) as a predictor of response, whilst belonging to the lower three (most deprived) quintiles of SIMD (OR 0.68 [95% CI 0.49, 0.95], *p* = 0.024) and older age at diagnosis (OR 0.973 [95% CI 0.966, 0.993] per year increment, *p* = 0.003) were independently associated with non-response (full model in ESM Table 5). In a separate model including low frequency of prior SMBG (<4/day), there was a borderline independent association with response (OR 1.70 [95% CI 0.99, 2.93], *p* = 0.055) (ESM Table 6).

### Questionnaire data

Frequency of symptomatic hypoglycaemia (defined as glucose <3.5 mmol/l) increased, with those reporting two to three episodes per week or more rising from 25.8% to 48.4% (*p* < 0.001) following flash monitor use (ESM Fig. 2). Similarly, the proportion of people experiencing any asymptomatic hypoglycaemia (<3.5 mmol/l) rose from 20.4% to 29.5% (*p* < 0.001) (ESM Fig. 3). No significant change in severe hypoglycaemia was observed (7.3% vs 8.8%, *p* = 0.499) although there was a non-significant increase in the number of episodes where unconsciousness or seizure occurred (2.1% vs 4.5%, *p* = 0.099). No significant difference was observed in Gold score (2 [IQR 1 to 2] vs 2 [1 to 3], *p* = 0.104) and the proportion of people with impaired awareness of hypoglycaemia (IAH, defined as Gold score ≥4) did not change (12.5% vs 13.1%, *p* = 0.867), although prior IAH resolved in 42.5% (17/40) of people following flash monitor use.

Administration of bolus insulin at least 15 min before meals increased from 9.3% to 36.2% following commencement of flash monitoring (*p* < 0.001).

Median HADS depression scores increased from 1 (IQR 0 to 3) to 2 (1 to 5, *p* < 0.001) with the proportion of those with an elevated score (>7) rising from 7.6% to 15.0% (*p* < 0.001). In the 8.2% of individuals (26/317) with newly elevated HADS depression score after flash monitoring commencement, the only difference observed was lower SIMD rank (2686 [IQR 1715 to 3959] vs 4857 [2884 to 6445], *p* < 0.001).

Median HADS anxiety scores increased from 4 (IQR 2 to 8) to 5 (2 to 8, *p* = 0.030) and the proportion with an elevated score rose from 24.9% to 30.9% (*p* = 0.028). HADS anxiety scores that were newly elevated after commencing flash monitoring developed in 12.3% (39/317) of people. This was associated with younger age (38 [IQR 26 to 47] vs 47 [35 to 58] years, *p* = 0.002), shorter duration of diabetes (13 [7 to 24] vs 22 [12 to 33] years, *p* = 0.002), prior history of self-funding (17.4% vs 9.2%, *p* = 0.031) and CSII use (21.7% vs 9.0%, *p* = 0.002).

These hypoglycaemia and HADS data were obtained from routinely collected clinic questionnaires. Paired pre- and post-flash monitoring questionnaires were available in 56.7% of eligible individuals (*n* = 334). Presence of paired questionnaire data was more likely in older individuals (45 years [IQR 33 to 57] vs 41 years [29 to 51], *p* = 0.001), those with lower baseline HbA_1c_ (63 mmol/mol [55 to 70] vs 65 mmol/mol [56 to 75], *p* = 0.023) (7.9% [7.2 to 8.6] vs 8.1% [7.3 to 9.00]) and prior DAFNE participants (62.8% vs 52.4%, *p* = 0.013) (ESM Table 7).

All individuals attending RIE flash monitoring education events were invited to complete a single online questionnaire which included a modified version of the DDS questionnaire, where potential responses were: ‘much more of a problem’ (assigned a value of −2), ‘more of a problem’ (value = −1), ‘unchanged’ (value = 0), ‘less of a problem’ (value = 1) and ‘much less of a problem’ (value = 2). The median responses were: 0 (IQR 0 to 0.3) for physician related distress, 0 (0 to 0.3) for interpersonal distress, 1 (0.4 to 1.4) for regimen-related distress and 0.6 (0.2 to 1.0) for the emotional component. Full results from the modified DDS are presented in ESM Table 8. The percentage of respondents with a net improvement in total DDS (90.0%), regimen-related distress (88.7%) and emotional distress (83.0%) were all significantly greater than 50.0% (*p* < 0.001). Respondents were also asked to provide a score from −5 (less control) to +5 (more control) with respect to the overall effect of flash monitoring on diabetes control; the median response was +5 (IQR 3 to 5). Of those who responded to the questionnaire, 70.6% reported taking their bolus insulin doses earlier in relation to meals following commencement of flash monitoring. The online questionnaire was completed by 54.1% of eligible individuals, with no demographic or diabetes-related differences between respondents and non-respondents (ESM Table 9).

### Flash monitoring data

When considering the entire cohort, where any flash monitoring data were available (*n* = 166), there were strong correlations with number of daily scans and both final HbA_1c_ (*r* −0.255, *p* < 0.001) and change in HbA_1c_ (*r* −0.279, *p* < 0.001). Paired flash monitoring data (i.e. within 7 days of first flash monitor use and at least 1 month later) was available in 53 of a possible 362 individuals with no prior self-funded flash monitor use. The only significant difference between those for whom this information was available and those for whom it was not was younger age at diagnosis (16 years [IQR 10 to 24] vs 21 years [12 to 33], *p* = 0.013) (ESM Table 10). The median interval between baseline and follow-up flash monitoring data was 6 months (IQR 5 to 7). No differences were observed in time between 3.9 and 10.0 mmol/l glucose, time above 10 mmol/l, time below 3.9 mmol/l, hypoglycaemic event number, hypoglycaemic event duration or mean glucose (ESM Table 11). The only significant difference was a fall in daily flash monitoring scans from baseline to follow-up (13 [IQR 9 to 19] vs 10 [6 to 14], *p* < 0.001).

### Hospital admission data

There was a significant reduction in admissions for diabetic ketoacidosis (DKA), falling from 10 to 2 episodes (*p* = 0.043) in the 6 months following NHS-funded flash monitoring (compared with the corresponding period 2 years earlier [2016]). No differences were noted in the 6 months following NHS-funded flash monitoring with respect to number of hospital admissions (*p* = 0.539), duration of hospital admissions (*p* = 0.680) or emergency department attendances (*p* = 0.449).

## Discussion

We have demonstrated a significant and clinically important reduction in HbA_1c_ in people with type 1 diabetes following flash monitor use. The clear temporal relationship between flash monitoring commencement and fall in HbA_1c_, supported by the absence of change in those who had not used flash monitoring, suggests flash monitor use as the causal factor. The magnitude of change in HbA_1c_ is consistent with previously published reports assessing CGM, such as the DIAMOND (−7 mmol/mol [0.6%]), GOLD (−5 mmol/mol [0.5%]) and JDRF (−6 mmol/mol [0.6%]) studies [12–14]. The HbA_1c_ change is also consistent with our previous small uncontrolled study of flash monitoring outcomes where the mean fall in HbA_1c_ was 5 mmol/mol (0.5%) [3].

No individual factor is sufficiently predictive to preclude the possibility of benefit in any particular group. However, these data clearly suggest the largest HbA_1c_ lowering benefits are observed in those with higher HbA_1c_. Prescribing guidance in the UK has often sought to limit flash monitor use to those who monitor blood glucose most frequently, however these data suggest this is illogical, as those who previously checked least frequently had significantly greater likelihood of achieving an HbA_1c_ fall of 5 mmol/mol or greater. The greater likelihood of HbA_1c_ response in those belonging to more affluent SIMD quintiles may represent a proxy for greater numeracy and data interpretation skills within this group [15], although it is difficult to reconcile why younger age at diagnosis but not age or duration of diabetes independently predicts response. As we have previously demonstrated [3], flash monitor use resulted in significant changes in bolus timing behaviour, which are typically associated with improvements in HbA_1c_ [16].

Self-reported episodes of both symptomatic and asymptomatic hypoglycaemia increased following flash monitor use. This is perhaps unsurprising as the additional glucose information provided by flash monitoring is likely to have highlighted hitherto unrecognised hypoglycaemia rather than implying the development of greater hypoglycaemia per se. The conventional wisdom that markedly increased hypoglycaemia is the expected trade-off for improved HbA_1c_ has been challenged by recent evidence assessing the impact of CGM [17]. Despite the additional information provided by flash monitoring, there was no significant improvement in awareness of hypoglycaemia. This study design cannot exclude the possibility that flash monitoring reduces rates of IAH, as it is possible that the 42.5% fall in those with pre-existing IAH represents genuine improvement in awareness, whilst some of the newly reported individuals with IAH may simply reflect greater recognition of previously unrecognised hypoglycaemia. The only head-to-head comparison of flash monitoring and CGM in IAH did, however, suggest benefit only with the latter technology, suggesting real-time alarms may be essential in this group [18].

Most investigations exploring the psychological impact of CGM (specific evidence for flash monitoring does not yet exist) have failed to demonstrate any significant change [19]. We found increased likelihood of elevated anxiety and depression scores following flash monitor use, although this conflicted with snapshot data from the DDS and user evaluation of the overall impact of flash monitoring, which was overwhelmingly positive. There do not appear to be any straightforward predictors of where depression and anxiety are likely to worsen following flash monitoring. Newly elevated depression score was associated with greater social deprivation (a recognised risk factor for depression [20]) but not any specific diabetes-related factors. The factors associated with newly elevated anxiety scores (younger age, shorter diabetes duration, prior flash monitoring self-funding and CSII use) may suggest a population with very high expectations for achieving tight glycaemic targets. These findings emphasise the importance of setting realistic targets and providing support to achieve them. The overall impression from user questionnaire feedback (including the DDS) is that commencement of flash monitoring has been a positive experience.

We chose to analyse only flash monitoring data from people with no prior history of flash monitor use and only when this was available within the first 7 days of use, as it is known that flash monitor data changes within days of commencement [4]. As this was a ‘real-world’ assessment, we did not have the benefit of any blinded flash monitoring data prior to commencement. Predictably, therefore, we did not find any differences in key glycaemic parameters. A previous study demonstrated a strong correlation between more flash monitoring scanning and lower estimated HbA_1c_ [21]; in addition, we have shown increased scanning is prospectively associated with fall in HbA_1c_.

A recent ‘real-world’ assessment demonstrated a significant reduction in hospitalisation following commencement of CGM [22], although this cohort included a very high prevalence of IAH. Overall, we were unable to detect any significant difference in total hospital admissions and emergency department attendances in the first 6 months after flash monitoring commencement; this may be explained by relatively low baseline admission rates in this young population. The number of individuals presenting with DKA was also small, but we did observe a significant reduction in hospital admissions for this indication. Although this is potentially important, caution should be exercised in extrapolating these findings to people with recurrent DKA admissions as this study cohort has different clinical and demographic characteristics [23].

Our study has a number of key strengths, most notably the cohort size which is, to our knowledge, the largest prospective assessment of flash monitoring outcomes described to date. We have also been able to present comprehensive longitudinal HbA_1c_ data, including a large comparator population with no flash monitoring, making the observed effects likely to be attributable to flash monitoring. Due to scrupulous recording of previous self-funded flash monitoring start dates, we have been able to avoid missing important early changes in HbA_1c_ in those who self-funded prior to NHS funding. Beyond HbA_1c_ results, we have been able to assess effects across a range of parameters important to individuals with diabetes (including hypoglycaemia and quality of life data), through routinely collected clinic data. Unlike many randomised controlled trials, this population is likely to be substantially more representative of usual clinical practice.

This study is open to the typical criticisms of observational methodology, in particular the possibility of unmeasured confounders and the potential for bias in relation to missing follow-up data. To limit the potential for bias we have assiduously reported the characteristics of those with incomplete follow-up data and find little to support the contention that this has significantly skewed the reported findings (i.e. no differences in factors important in predicting fall in HbA_1c_). Arguably a coincident advance in diabetes care could have accounted for the observed improvement but no such change was introduced to our centre in the past 2 years, and whole clinic data clearly demonstrate the improvement in HbA_1c_ being limited to flash monitor users. The findings of this study are not necessarily generalisable to everyone with type 1 diabetes, as we report significant differences in the characteristics of flash monitor users and non-users. However, given our relatively liberal eligibility criteria for flash monitoring commencement, they are likely to be generalisable to the population of people who would seek to use flash monitoring.

## Conclusions

Flash monitoring is associated with substantial reductions in HbA_1c_, particularly in those with higher HbA_1c_ prior to use. Although user satisfaction is very high, there are potentially issues around hypoglycaemia and psychological distress, at least in the early phase of treatment intensification. The observed fall in DKA admissions is promising and requires validation in larger cohorts. It is essential that evidence gathering in the field of glucose monitoring is responsive to the rapid and accelerating rate of change, to ensure potential benefits are realised early and by the largest possible number of people.

## Electronic supplementary material

ESM (PDF 1064 kb)

## Data Availability

The datasets generated during and/or analysed during the current study are available from the corresponding author on reasonable request. However, as this is a clinical dataset, the ability to share data is limited by patient confidentiality considerations.

## Abbreviations

CGM: Continuous glucose monitoring
CSII: Continuous subcutaneous insulin infusion
DAFNE: Dose Adjustment for Normal Eating
DDS: Diabetes distress scale
DKA: Diabetic ketoacidosis
HADS: Hospital Anxiety and Depression Scale
IQR: Interquartile range
IAH: Impaired awareness of hypoglycaemia
MDI: Multiple daily injection
NHS: National Health Service
NICE: National Institute for Health and Care Excellence
RIE: Royal Infirmary of Edinburgh
SIMD: Scottish Index of Multiple Deprivation
SMBG: Self-monitoring of blood glucose

## References

[1] Leelarathna L, Wilmot EG (2018). Flash forward: a review of flash glucose monitoring. Diabet Med.

[2] Gibb FW, McKnight JA (2017). Flash glucose monitoring is associated with improved glycaemic control but use is largely limited to more affluent people in a UK diabetes centre. Diabet Med.

[3] Dover AR, Stimson RH, Zammitt NN, Gibb FW (2016). Flash glucose monitoring improves outcomes in a type 1 diabetes clinic. J Diabetes Sci Technol.

[4] Bolinder J, Antuna JR, Geelhoed-Duijvestijn P, Kröger J, Weitgasser R (2016). Novel glucose-sensing technology and hypoglycaemia in type 1 diabetes: a multicentre, non-masked, randomised controlled trial. Lancet.

[5] Edinburgh Centre for Endocrinology and Diabetes (2018) Libre Talk – Royal Infirmary of Edinburgh. Available from www.edinburghdiabetes.com/libretalk. Accessed 23 Jan 2019

[6] National Institution for Health and Care Excellence (2015) Type 1 diabetes in adults: diagnosis and management. Available from www.nice.org.uk/guidance/ng17. Accessed 23 Jan 2019

[7] Scottish Government. Scottish Index of Multiple Deprivation (2016) Available from www2.gov.scot/Topics/Statistics/SIMD. Accessed 23 Jan 2019

[8] DAFNE Study Group (2002). Training in flexible, intensive insulin management to enable dietary freedom in people with type 1 diabetes: dose adjustment for normal eating (DAFNE) randomised controlled trial. BMJ.

[9] Geddes J, Wright RJ, Zammitt NN, Deary IJ, Frier BM (2007). An evaluation of methods of assessing impaired awareness of hypoglycaemia in type 1 diabetes. Diabetes Care.

[10] Zigmond AS, Snaith RP (1983). The hospital anxiety and depression scale. Acta Psychiatr Scand.

[11] Polonsky WH, Fisher L, Earles J (2005). Assessing psychosocial distress in diabetes: development of the diabetes distress scale. Diabetes Care.

[12] Beck RW, Riddlesworth T, Ruedy K (2017). Effect of continuous glucose monitoring on glycemic control in adults with type 1 diabetes using insulin injections: the DIAMOND randomised clinical trial. JAMA.

[13] Lind M, Polonsky W, Hirsch IB (2017). Continuous glucose monitoring vs conventional therapy for glycemic control in adults with type 1 diabetes treated with multiple daily insulin injections: the GOLD randomised clinical trial. JAMA.

[14] Tamborlane WV, Beck RW, Juvenile Diabetes Research Foundation Continuous Glucose Monitoring Study Group (2008). Continuous glucose monitoring and intensive treatment of type 1 diabetes. N Engl J Med.

[15] Marden S, Thomas PW, Sheppard ZA, Knott J, Lueddeke J, Kerr D (2012). Poor numeracy skills are associated with glycaemic control in type 1 diabetes. Diabet Med.

[16] Slattery D, Amiel SA, Choudhary P (2017). Optimal prandial timing of bolus insulin in diabetes management: a review. Diabet Med.

[17] Gimenez M, Tannen AJ, Reddy M, Moscardo V, Conget I, Oliver N (2018). Revisiting the relationships between measures of glycemic control and hypoglycaemia in continuous glucose monitoring data sets. Diabetes Care.

[18] Reddy M, Jugnee N, Anantharaja S, Oliver N (2018). Switching from flash glucose monitoring to continuous glucose monitoring on hypoglycaemia in adults with type 1 diabetes at high hypoglycaemia risk: the extension phase of the I HART CGM study. Diabetes Technol Ther.

[19] Patton SR, Clements MA (2016). Psychological reactions associated with continuous glucose monitoring in youth. J Diabetes Sci Technol.

[20] Ostler K, Thompson C, Kinmonth AK, Peveler RC, Stevens L, Stevens A (2001). Influence of socio-economic deprivation on the prevalence and outcome of depression in primary care. Br J Psychiatry.

[21] Dunn TC, Xu Y, Hayter G, Ajjan R (2017). Real-world flash glucose monitoring patterns and associations between self-monitoring frequency and glycaemic measures: a European analysis of over 60 million glucose tests. Diabetes Res Clin Pract.

[22] Charleer S, Mathieu C, Nobels F (2018). Effect of continuous glucose monitoring on glycemic control, acute admissions, and quality of life: a real-world study. J Clin Endocrinol Metab.

[23] Gibb FW, Teoh WL, Graham J, Lockman KA (2016). Risk of death following admission to a UK hospital with diabetic ketoacidosis. Diabetologia.

