# Supplementary material for: Marked improvement in HbA1c following commencement of flash glucose monitoring in people with type 1 diabetes
Source: Diabetologia. 2019 Jun 9;62(8):1349–56. doi: 10.1007/s00125-019-4894-1 (PMC6647076; doi:10.1007/s00125-019-4894-1)
Supplement: Supplementary file 1 — (PDF 1064 kb) [file 125_2019_4894_MOESM1_ESM.pdf]

**ESM table 1:** Baseline characteristics (comparing the two participating clinics). FM, flash monitoring

| | RIE | WGH | Total |
|---|---|---|---|
| n | 589 | 311 | 900 |
| Sex | 279 (47.3%) F / 310 (52.7%) M | 160 (51.4%) F / 151 (48.6%) M | 439 (48.8%) F / 461 (51.2%) M |
| Age at FM commencement (years) | 43 (31 – 54) | 40 (30 – 55) | 42 (30 – 55) |
| Age at diagnosis (years) | 19 (11 – 30) | 19 (12 – 31) | 19 (12 – 31) |
| Duration of diabetes (years) | 21 (11 – 31) | 17 (8 – 28) | 19 (10 – 30) |
| Previous self-funded FM use | 227 (38.5%) | 127 (40.8%) | 354 (39.3%) |
| Last HbA1c prior to any Libre use (self-funders) (mmol/mol & %) | 64 (56 – 73)<br>8.0% (7.3 – 8.8) | 63 (54 – 71)<br>7.9% (7.1 – 8.6) | 63 (55 – 73)<br>7.9% (7.2 – 8.8) |
| Last HbA1c prior to NHS-funded FM (non-self-funders) (mmol/mol & %) | 63 (55 – 72)<br>7.9% (7.2 – 8.7) | 62 (54 – 72)<br>7.8% (7.1 – 8.7) | 63 (55 – 72)<br>7.9% (7.2 – 8.7) |
| Last HbA1c prior to NHS-funded FM use (self-funders) (mmol/mol & %) | 63 (56 – 72)<br>7.9% (7.3 – 8.7) | 62 (54 – 71)<br>7.8% (7.1 – 8.6) | 63 (55 – 72)<br>7.9% (7.2 – 8.7) |
| Last HbA1c prior to NHS-funded FM use (all) (mmol/mol & %) | 62 (54 – 71)<br>7.8% (7.1 – 8.6) | 61 (52 – 71)<br>7.7% (6.9 – 8.6) | 61 (53 – 71)<br>7.7% (7.0 – 8.6) |
| Achieving Scottish HbA1c target (<58 mmol/mol) | 31.3% | 36.4% | 33.1% |
| Achieving NICE HbA1c target (≤ 48 mmol/mol) | 8.9% | 11.4% | 9.8% |
| BMI prior to FM use (kg/m$^2$) | 26.0 (23.4 – 29.0) | NA | NA |
| SIMD 2016 rank (out of 6976) | 4705 (2697 – 6398) | 5579 (3671 – 6566) | 5063 (3071 – 6493) |
| CSII | 158 (26.9%) | 84 (27.0%) | 242 (26.9%) |
| DAFNE | 242 (41.1%) | 89 (28.6%) | 331 (36.8%) |

**ESM table 2:** Differences between those with paired HbA1c measurements and those where paired HbA1c was not available. *'Self-funder' refers to any previous self-funded flash monitor use prior to NHS-funding becoming available.

| | Paired HbA1c available (n = 743) | No paired HbA1c available (n = 157) | P |
|---|---|---|---|
| Age (years) | 42 (31 – 55) | 41 (29 – 52) | 0.109 |
| Duration of diabetes (years) | 19 (10 – 30) | 18 (10 – 30) | 0.475 |
| Age at diagnosis (years) | 19 (12 – 30) | 18 (12 – 31) | 0.775 |
| Baseline HbA1c (mmol/mol) | 63 (55 – 72) | 65 (57 – 74) | 0.054 |
| Baseline HbA1c (%) | 7.9 (7.2 – 8.7) | 8.1 (7.4 – 8.9) | 0.054 |
| SIMD rank (out of 6976) | 5025 (3071 – 6493) | 5089 (3436 – 6454) | |
| **Female** | **85.6%** | **14.4%** | |
| **Male** | **79.6%** | **20.4%** | **0.017** |
| **RIE** | **78.3%** | **21.7%** | |
| **WGH** | **90.7%** | **9.3%** | **< 0.001** |
| Self-funder* | 82.2% | 17.8% | |
| Non self-funder | 82.8% | 17.2% | 0.822 |
| DAFNE yes | 84.9% | 15.1% | |
| DAFNE no | 81.2% | 18.8% | 0.159 |
| **MDI** | **80.7%** | **19.3%** | |
| **CSII** | **87.6%** | **12.4%** | **0.016** |

**ESM table 3:** Longitudinal HbA1c (mmol/mol and %) by flash monitoring category. Data are median (IQR).

| | **Self-fund (n = 162)** | **NHS FM (n = 250)** | **Late NHS FM (n = 153)** | **No FM (n = 518)** |
|---|---|---|---|---|
| 2014 | 60 (52 – 68)<br>7.6 (6.9 – 8.4) | 59 (53 – 67)<br>7.5 (7.0 – 8.3) | 63 (54 – 75)<br>7.9 (7.1 – 9.0) | 63 (56 – 72)<br>7.9 (7.3 – 8.7) |
| 2015 | 58 (52 – 67)<br>7.5 (6.9 – 8.3) | 60 (53 – 68)<br>7.6 (7.0 – 8.4) | 64 (55 – 76)<br>8.0 (7.2 – 9.1) | 64 (57 – 72)<br>8.0 (7.4 – 8.7) |
| 2016 | 61 (54 – 69)<br>7.7 (7.1 – 8.5) | 62 (55 – 71)<br>7.8 (7.2 – 8.6) | 66 (56 – 76)<br>8.2 (7.3 – 9.1) | 65 (57 – 74)<br>8.1 (7.4 – 8.9) |
| 2017 | 61 (53 – 67)<br>7.7 (7.0 – 8.3) | 62 (55 – 71)<br>7.8 (7.2 – 8.6) | 66 (54 – 74)<br>8.2 (7.1 – 8.9) | 65 (57 – 76)<br>8.1 (7.4 – 9.1) |
| 2018 | 56 (49 – 63)<br>7.3 (6.6 – 7.9) | 58 (51 – 66)<br>7.5 (6.8 – 8.2) | 63 (53 – 72)<br>7.9 (7.0 – 8.7) | 65 (57 – 75)<br>8.1 (7.4 – 9.0) |
| P for comparison between 2016 and 2018 | < 0.001 | < 0.001 | 0.008 | 0.508 |

**Self-fund:** individuals who self-funded purchase of FM prior to taking up NHS-funded sensors in Feb/Mar 2018. **NHS FM:** individuals whose first FM use was in Feb/Mar 2018 (i.e. no self-funded use). **Late NHS FM:** Individuals whose first FM use was after Mar 2018 (i.e. no self-funded use). **No FM:** Individuals with no previous or current FM use. FM, flash monitor

**ESM table 4:** Univariate analysis of individuals who achieved 5 mmol/mol or greater decline in HbA1c and those who did not. Data are median (IQR).

| | Fall of 5 mmol/or more (n = 357) | No fall of 5 mmol/mol (n = 382) | P |
|---|---|---|---|
| Age (years) | 42.3 (30.9 – 53.3) | 42.4 (30.9 – 56.4) | 0.525 |
| **Age at diagnosis (years)** | **18.0 (11.1 – 28.6)** | **20.3 (11.7 – 23.5)** | **0.032** |
| Duration of diabetes (years) | 20.1 (10.8 – 30.8) | 18.1 (9.0 – 29.9) | 0.132 |
| Male | 170 (46.8%) | 193 (53.2%) | |
| Female | 187 (49.7%) | 189 (50.3%) | 0.430 |
| Self-funder | 150 (51.7%) | 140 (48.3%) | |
| Non self-funder | 207 (46.1%) | 242 (53.9%) | 0.135 |
| **Baseline HbA1c (mmol/mol)** | **68 (60 – 78)** | **58 (51 – 65)** | **< 0.001** |
| **Baseline HbA1c (%)** | **8.4 (7.6 – 9.3)** | **7.5 (6.8 – 8.1)** | **< 0.001** |
| SIMD quintiles 1- 3 (more deprived) | 132 (46.5%) | 152 (53.5%) | |
| SIMD quintiles 4 – 5 (less deprived) | 216 (49.4%) | 221 (50.6%) | 0.439 |
| CSII | 99 (46.9%) | 112 (53.1%) | |
| MDI | 258 (48.9%) | 270 (51.1%) | 0.632 |
| DAFNE yes | 133 (47.7%) | 146 (52.3%) | |
| DAFNE no | 224 (48.7%) | 236 (51.3%) | 0.787 |
| **Prior SMBG less than 4 times / day** | **67 (67.7%)** | **32 (32.3%)** | |
| **Prior SMBG 4 times or more / day** | **131 (45.2%)** | **159 (54.8%)** | **< 0.001** |

**ESM table 5:** Logistic regression analysis for HbA1c decline of 5 mmol/mol or greater.

| n = 717 | B (SE) | Lower CI | OR | Upper CI | P |
|---|---|---|---|---|---|
| Intercept | -4.091 (0.517) | | | | |
| Age (years) | 0.009 (0.006) | 0.997 | 1.009 | 1.022 | 0.132 |
| **Age at diagnosis (years)** | **-0.021 (0.007)** | **0.966** | **0.973** | **0.993** | **0.003** |
| **Baseline HbA1c (mmol/mol)** | **0.066 (0.007)** | **1.054** | **1.068** | **1.084** | **<0.001** |
| **In quintiles 1 -3 SIMD 2016** | **-0.383 (0.170)** | **0.487** | **0.681** | **0.950** | **0.024** |

**ESN table 6:** Logistic regression analysis for HbA1c decline of 5 mmol/mol or greater (including SBGM data – reduced dataset).

| n = 381 | B (SE) | Lower CI | OR | Upper CI | P |
|---|---|---|---|---|---|
| Intercept | -4.306 (0.767) | | | | |
| Age (years) | 0.008 (0.009) | 0.990 | 1.008 | 1.025 | 0.398 |
| **Age at diagnosis (years)** | **-0.022 (0.010)** | **0.958** | **0.978** | **0.998** | **0.035** |
| **Baseline HbA1c (mmol/mol)** | **0.073 (0.011)** | **1.054** | **1.076** | **1.100** | **<0.001** |
| **In quintiles 1 -3 SIMD 2016** | **-0.721 (0.235)** | **0.305** | **0.486** | **0.767** | **0.002** |
| Fewer than 4 blood glucose tests/day at baseline | 0.528 (0.275) | 0.992 | 1.695 | 2.926 | 0.055 |

**ESM table 7:** Differences between those with paired HbA1c questionnaire data and those where this was not available.

| | Paired questionnaire available (n = 334) | No paired questionnaire (n = 255) | P |
|---|---|---|---|
| **Age (years)** | **45 (33 – 57)** | **41 (29 – 51)** | **0.001** |
| Duration of diabetes (years) | 22 (11 – 33) | 20 (10 – 29) | 0.060 |
| Age at diagnosis (years) | 19 (11 – 32) | 18 (11 – 28) | 0.151 |
| **Baseline HbA1c (mmol/mol)** | **63 (55 – 70)** | **65 (56 – 75)** | **0.023** |
| **Baseline HbA1c (%)** | **7.9 (7.2 – 8.6)** | **8.1 (7.3 – 9.0)** | **0.023** |
| Change in hbA1c pre- to post- FM (mmol/mol) | -5 (-10 – -1) | -5 (-10 – 1) | 0.902 |
| Change in hbA1c pre- to post- FM (%) | -0.46 (-0.91 – -0.09) | -0.46 (-0.91 - -0.09) | 0.902 |
| SIMD rank (out of 6976) | 4547 (2660 – 6358) | 4766 (2788 – 6418) | 0.383 |
| Female | 165 (59.1%) | 114 (40.9%) | |
| Male | 169 (54.5%) | 141 (54.5%) | 0.258 |
| Self-funder | 129 (56.8%) | 98 (43.2%) | |
| Non self-funder | 205 (56.6%) | 157 (43.4%) | 0.962 |
| **DAFNE yes** | **152 (62.8%)** | **90 (37.2%)** | |
| **DAFNE no** | **182 (52.4%)** | **165 (47.6%)** | **0.012** |
| MDI | 245 (56.8%) | 186 (43.2%) | |
| CSII | 89 (56.3%) | 69 (43.7%) | 0.986 |

**ESM table 8:** Responses to individual components of the modified Diabetes Distress Scale following FM use (n = 318 of 589 invited). Most common response marked in **bold**.

| | Much more of a problem | More of a problem | No change | Less of a problem | Much Less of a problem |
|---|---|---|---|---|---|
| | Emotional Burden | | | | |
| E1 | 0.0% | 6.0% | 22.1% | **48.6%** | 23.3% |
| E2 | 0.0% | 4.4% | **49.8%** | 36.3% | 9.5% |
| E3 | 0.0% | 5.4% | 38.5% | **43.8%** | 12.3% |
| E4 | 0.3% | 3.8% | 39.2% | **42.1%** | 14.6% |
| E5 | 0.9% | 7.9% | **43.7%** | 36.1% | 11.4% |
| | Regimen-related Distress | | | | |
| R1 | 0.0% | 1.0% | 15.9% | 25.4% | **57.8%** |
| R2 | 1.3% | 6.0% | 21.4% | **50.6%** | 20.8% |
| R3 | 0.3% | 5.0% | 20.5% | **47.9%** | 26.2% |
| R4 | 1.6% | 10.2% | **48.9%** | 29.5% | 9.8% |
| R5 | 0.0% | 1.0% | 33.0% | **42.2%** | 23.8% |
| | Interpersonal distress | | | | |
| I1 | 0.0% | 0.9% | **69.0%** | 19.0% | 11.1% |
| I2 | 0.9% | 3.5% | **74.7%** | 15.5% | 5.4% |
| I3 | 0.3% | 2.2% | **84.2%** | 8.2% | 5.1% |
| | Physician-related distress | | | | |
| P1 | 1.0% | 2.6% | **73.7%** | 15.4% | 7.4% |
| P2 | 0.6% | 2.5% | **73.7%** | 16.5% | 6.7% |
| P3 | 0.3% | 1.3% | **85.4%** | 8.3% | 4.8% |
| P4 | 2.5% | 6.4% | **74.8%** | 10.5% | 5.7% |

**Key to questions:**

**E1** Feeling that diabetes is taking up too much of my mental and physical energy every day **E2** Feeling angry, scared, and/or depressed when I think about living with diabetes **E3** Feeling that diabetes controls my life **E4** Feeling that I will end up with serious long-term complications, no matter what I do **E5** Feeling overwhelmed by the demands of living with diabetes

**R1** Feeling that I am not testing my blood sugars frequently enough **R2** Feeling that I am often failing with my diabetes routine **R3** Not feeling confident in my day-to-day ability to manage diabetes **R4** Feeling that I am not sticking closely enough to a good meal plan **R5** Not feeling motivated to keep up my diabetes self-management

**I1** Feeling that friends or family are not supportive enough of self-care efforts (e.g. planning activities that conflict with my schedule, encouraging me to eat the "wrong" foods) **I2** Feeling that friends or family don't appreciate how difficult living with diabetes can be **I3** Feeling that friends or family don't give me the emotional support that I would like

**P1** Feeling that my doctor doesn't know enough about diabetes and diabetes care **P2** Feeling that my doctor doesn't give me clear enough directions on how to manage my diabetes **P3** Feeling that my doctor doesn't take my concerns seriously enough **P4** Feeling that I don't have a doctor who I can see regularly enough about my diabetes

**ESM table 9:** Differences between those with online questionnaire data and those where this was not available.

| | Online questionnaire data available (n = 319) | No online questionnaire (n = 270) | P |
|---|---|---|---|
| Age (years) | 43 (32 – 55) | 42 (30 – 54) | 0.586 |
| Duration of diabetes (years) | 22 (12 – 31) | 20 (10 – 31) | 0.516 |
| Age at diagnosis (years) | 19 (12 – 29) | 19 (10 – 32) | 0.704 |
| Baseline HbA1c (mmol/mol) | 63 (56 – 71) | 64 (56 – 74) | 0.413 |
| Baseline HbA1c (%) | 7.9 (7.3 – 8.6) | 8.0 (7.3 – 8.9) | 0.413 |
| Change in hbA1c pre- to post- FM (mmol/mol) | -5 (-10 – -1) | -4 (-10 – 0) | 0.068 |
| Change in hbA1c pre- to post- FM (%) | -0.46 (-0.91 - -0.09) | -0.37 (-0.91 – 0) | 0.068 |
| SIMD rank (out of 6976) | 4820 (2834 – 6450) | 4478 (2657 – 6279) | 0.357 |
| Female | 142 (50.9%) | 137 (49.1%) | |
| Male | 177 (57.1%) | 133 (42.9%) | 0.131 |
| Self-funder | 130 (57.3%) | 97 (42.7%) | |
| Non self-funder | 189 (52.2%) | 173 (47.8%) | 0.230 |
| DAFNE yes | 127 (52.5%) | 115 (47.5%) | |
| DAFNE no | 192 (55.3%) | 155 (47.5%) | 0.494 |
| MDI | 233 (54.1%) | 198 (45.9%) | |
| CSII | 86 (54.4%) | 72 (45.6%) | 0.936 |

**ESM table 10:** Differences between those with paired FM data and those where this was not available. FM, flash monitor

| | Paired FM data available (n = 53) | No paired FM data available (n = 309) | P |
|---|---|---|---|
| Age (years) | 40 (30 – 52) | 47 (33 – 57) | 0.069 |
| Duration of diabetes (years) | 22 (12 – 35) | 21 (11 – 31) | 0.593 |
| **Age at diagnosis (years)** | **16 (10 – 24)** | **20 (12 – 33)** | **0.013** |
| Baseline HbA1c (mmol/mol) | 62 (56 – 67) | 64 (55 – 72) | 0.289 |
| Baseline HbA1c (%) | 7.8 (7.3 – 8.3) | 8.0 (7.2 – 8.7) | 0.289 |
| Change in hbA1c pre- to post- FM (mmol/mol) | -6 (-11 – -1) | -4 (-9 – 0) | 0.162 |
| Change in hbA1c pre- to post- FM (%) | -0.55 (-1.01 - -0.09) | -0.37 (-0.82 – 0) | 0.162 |
| SIMD rank (out of 6976) | 26 (24 – 30) | 26 (23 – 29) | 0.842 |
| Female | 31 (18.2%) | 139 (81.8%) | |
| Male | 22 (11.5%) | 170 (88.5%) | 0.069 |
| DAFNE yes | 18 (12.6%) | 125 (87.4%) | |
| DAFNE no | 35 (16.0%) | 184 (84.0%) | 0.372 |
| MDI | 39 (13.5%) | 250 (86.5%) | |
| CSII | 14 (19.2%) | 59 (80.8%) | 0.220 |

**ESM table 11:** Comparison of FM data commencing within first week of use and at follow-up (at least one month later) in people with no prior FM use. Based on 14-day glucose profile data. FM, flash monitor

| n = 53 | Early-FM | Follow-up | P |
|---|---|---|---|
| % time in range (3.9 – 10 mmol/L) | 54 (44 – 64) | 51 (41 – 64) | 0.545 |
| % time above range | 41 (31 – 52) | 41 (26 – 54) | 0.826 |
| % time below range | 4 (2 – 6) | 4 (2 – 10) | 0.157 |
| Number of low glucose (<3.9 mmol/L) events | 8 (3 – 13) | 8 (3 – 15) | 0.971 |
| Mean duration of low glucose events (minutes) | 91 (60 – 108) | 96 (67 – 135) | 0.191 |
| Mean glucose (mmol/L) | 9.5 (8.7 – 10.8) | 9.7 (8.2 – 11.0) | 0.954 |
| Estimated HbA1c (mmol/mol)<br>Estimated HbA1c (%) | 60 (54 – 68)<br>7.6 (7.1 – 8.4) | 60 (54 – 69)<br>7.6 (7.1 – 8.5) | 0.877 |
| **Daily FM scans performed** | **13 (9 – 19)** | **10 (6 – 14)** | **< 0.001** |

**ESM figure 1:** Relationship between interval from baseline to next HbA1c measurement and change in HbA1c (baseline to after flash monitoring). R 0.044, P = 0.244

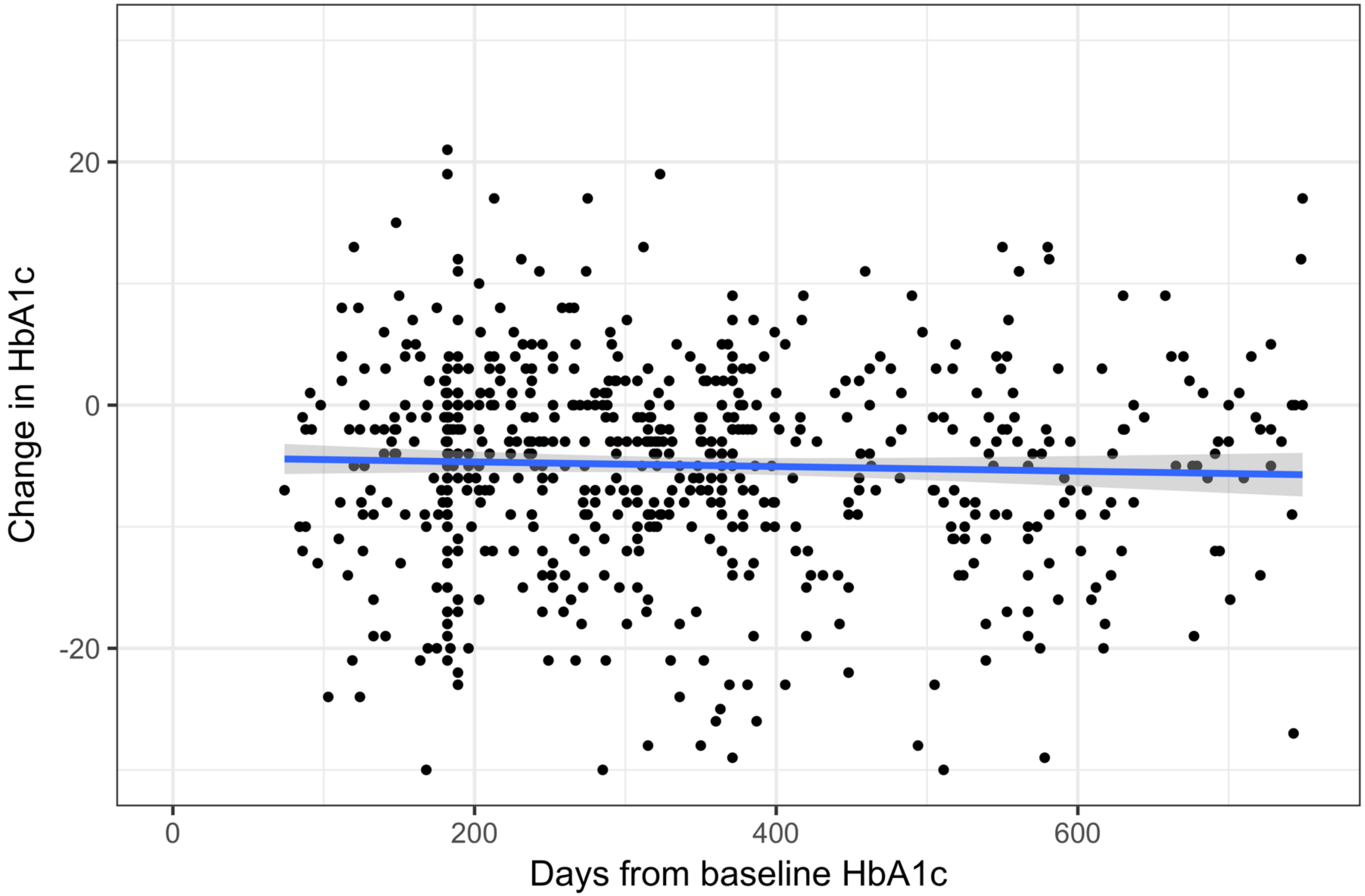

**ESM figure 2:** Symptomatic hypoglycemia rate before and after flash monitoring (n = 334). FM, flash monitoring

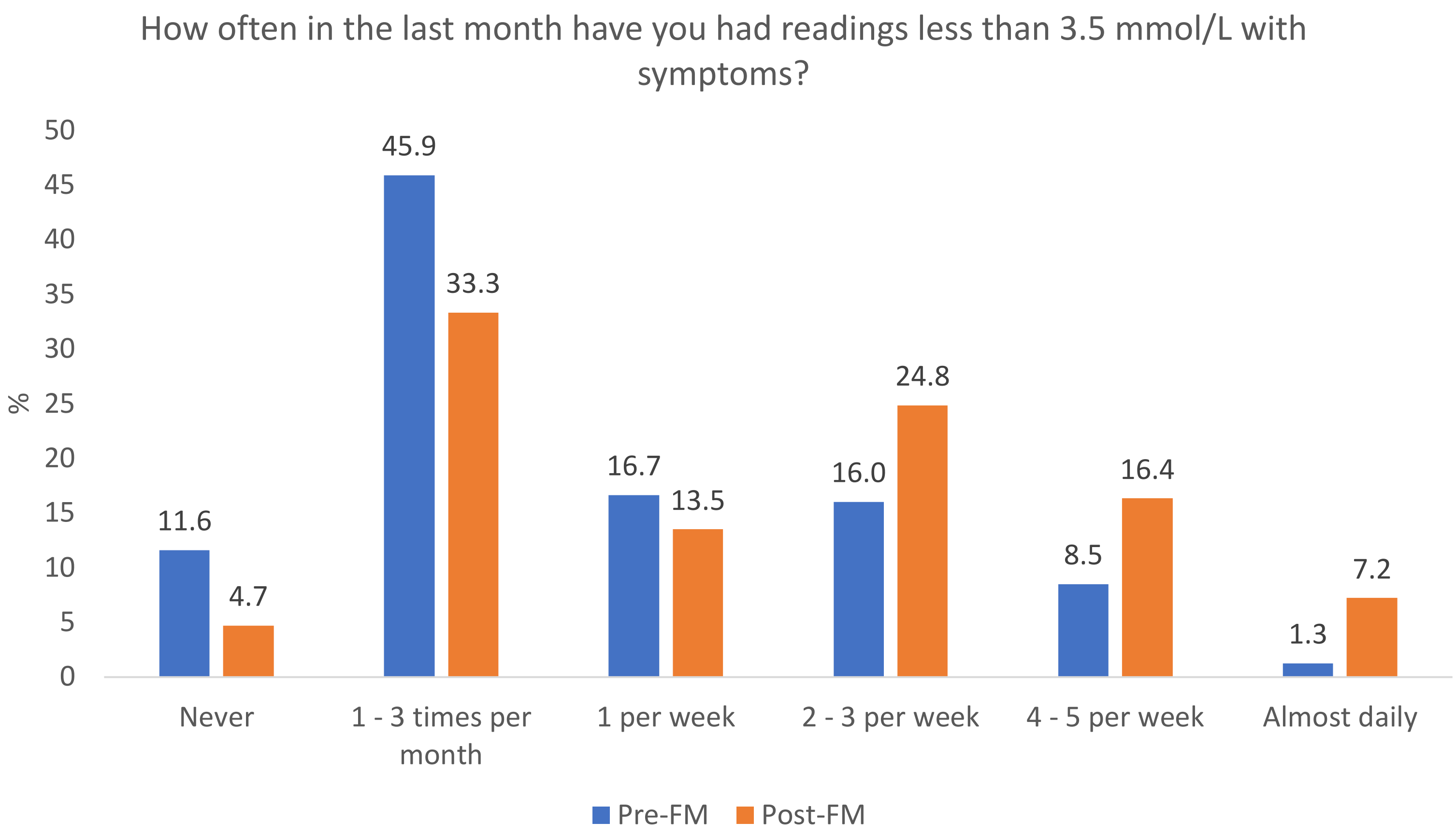

**ESM figure 3:** Asymptomatic hypoglycemia rate before and after flash monitoring (n = 334). FM, flash monitoring

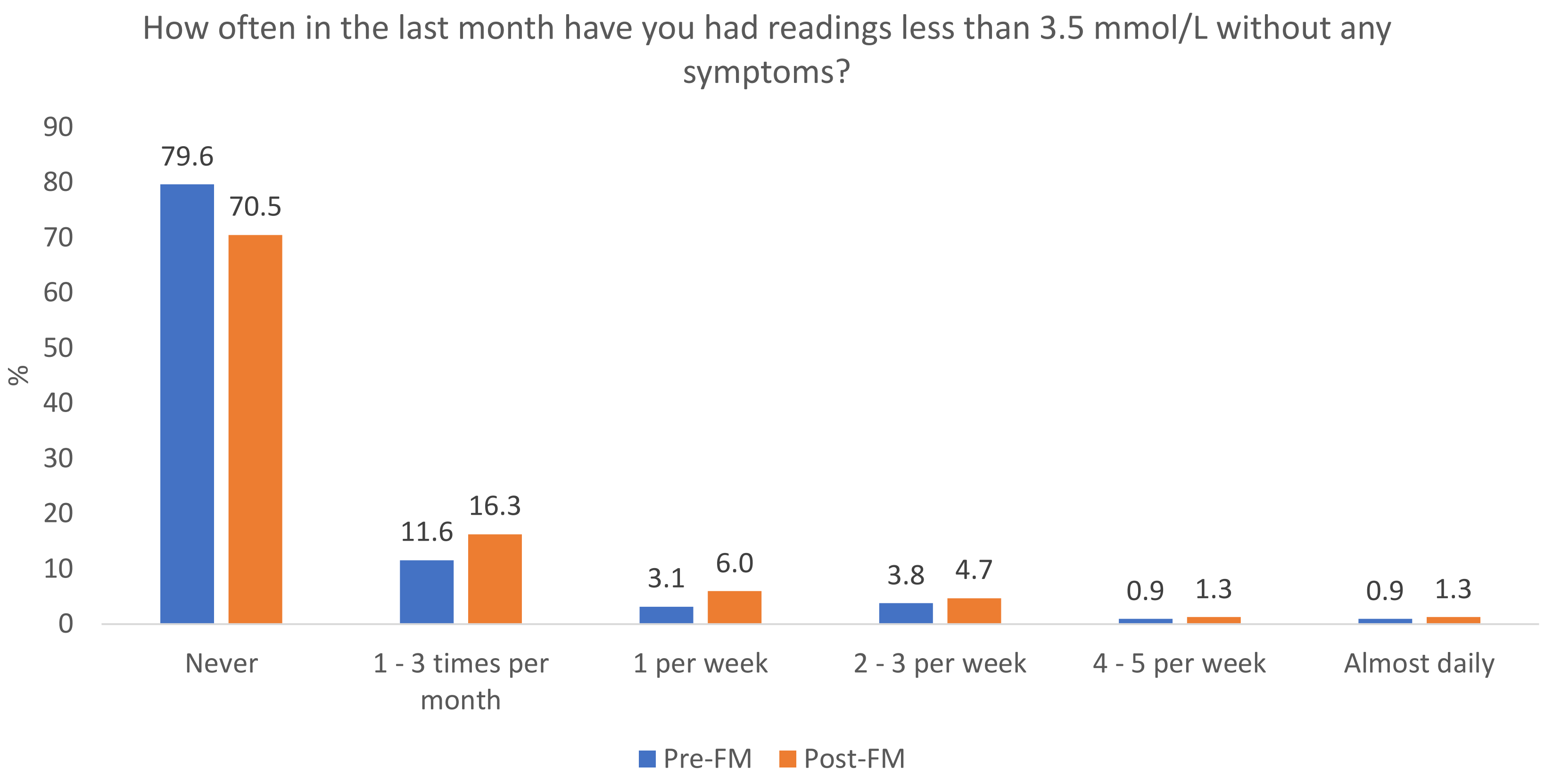

**ESM questionnaire 1**

www.edinburghdiabetes.com

## Improving diabetes care

## Royal Infirmary of Edinburgh

Name: ______________________________ Date: ______________

In order to get the most out of your diabetes clinic consultation, we would be grateful if you could complete this brief set of questions, each time you attend the clinic. Please hand the completed form to your clinic doctor.

Are there any specific issues which you would like to discuss during your appointment today?

[ ]

Some common topics are listed on the back page

If you would like to access your diabetes clinic results and letters, please provide your email address (this will give you access to the 'my diabetes my way' website):

@

1. Please fill in the types of insulin you are taking and the typical doses (or the approximate range of doses if these vary day-to-day):

| | Morning | Lunch | Evening meal | Before bed |
|---|---|---|---|---|
| Insulin name: | units | units | units | units |
| Insulin name: | units | units | units | units |

2. a. Where do you inject your insulin? [ ]

   b. What size of needles do you use? [ ] mm or Don't know [ ]

   c. Are you having any problems with your insulin injection sites? Yes [ ] No [ ]

3. Do you 'carbohydrate [carb] count'?

   Yes [ ] — Go to question 4

   No [ ] — Go to question 6

   Don't know [ ] — Go to question 6

4. What are your insulin to carbohydrate ratios (*e.g.* 1 unit for 10 grams carbohydrate [1 CP])

| Breakfast | Lunch | Evening meal |
|---|---|---|
| | | |

Please turn over...

5. What is your 'correction factor' (i.e. How much does 1 unit of insulin lower your blood sugar)?

Correction factor: or Don't know ☐

6. When do you usually (*i.e.* most often) take your quick acting insulin?

15 – 20 minutes before meals ☐ Just before meals ☐ With meals ☐ After meals ☐

7. How often do you <u>typically</u> check your blood sugar levels?

More than 4 times per day ☐ 4 times per day ☐ 3 times per day ☐ 2 times per day ☐ 1 time per day ☐

**8. Hypoglycaemia (low blood sugar) questions**

a. Tick the category that best describes you (tick **one** only):

I always have symptoms when my blood sugar is low ☐

I sometimes have symptoms when my blood sugar is low ☐

I no longer have symptoms when my blood sugar is low ☐

b. Have you lost some of the symptoms that used to occur when your blood sugar was low?

Yes ☐ No ☐

c. In the past 6 months, how often have you had hypoglycaemic episodes, where you might feel confused, disorientated, or lethargic **<u>AND WERE UNABLE TO TREAT YOURSELF</u>**?

Never ☐ Once or twice ☐ Every other month ☐

Once a month ☐ More than once a month ☐

d. In the past year, how often have you had hypoglycaemic episodes, where you were **unconscious** or had a **seizure** and needed glucagon or intravenous glucose?

Never ☐ 1 time ☐ 2 times ☐

3 – 5 times ☐ 5 – 10 times ☐ 10 or more times ☐

e. How often in the last month have you had readings less than 3.5 mmol/L <u>with symptoms</u>

Never ☐ 1 – 3 times ☐ 1 time per week ☐

2 – 3 times per week ☐ 4 – 5 times per week ☐ Almost daily ☐

Questionnaire continues >

f. How often in the last month have you had readings less than 3.5 mmol/L **without** any symptoms?

Never ☐ 1 – 3 times ☐ 1 time per week ☐

2 – 3 times per week ☐ 4 – 5 times per week ☐ Almost daily ☐

g. How low does you blood sugar need to go before you feel symptoms?

3.4 – 3.9 mmol/L ☐ 2.8 – 3.3 mmol/L ☐ 2.2 – 2.7 mmol/L ☐ Less than 2.2 mmol/L ☐

h. To what extent can you tell by your symptoms that your blood sugar is low?

Never ☐ Rarely ☐ Sometimes ☐ Often ☐ Always ☐

i. Do you always know when your hypos are commencing? Please circle a number.

Always aware 1 2 3 4 5 6 7 Never aware

---

9. Do you have a driving licence?

Yes ☐ Go to question 10 No ☐ Go to question 11

---

10. Prior to driving, do you check your blood sugar level and ensure it is above 5 mmol/L? Yes ☐ No ☐

When driving, do you keep short-acting carbohydrate and a blood sugar meter in your car? Yes ☐ No ☐

If your blood sugar falls below 4 mmol/L, do you wait at least 45 minutes after it is above 5 mmol/L before driving again? Yes ☐ No ☐

---

11. Do you smoke? Yes ☐ No ☐

---

12. **Question for male patients**
In the past year, have you experienced any difficulty in achieving or maintaining an erection? Yes ☐ No ☐

Prefer not to answer ☐

Please turn over...

## 13. General well-being questions

Please <u>**circle**</u> the number beside the response closest to how you have been feeling in the last week for each of the 14 questions. Don't think about it for too long – your immediate response is best.

| D | A | |
|---|---|---|
| | | **I feel tense or 'wound up':** |
| | 3 | Most of the time |
| | 2 | A lot of the time |
| | 1 | From time to time, occasionally |
| | 0 | Not at all |
| | | |
| | | **I still enjoy the things I used to enjoy:** |
| 0 | | Definitely as much |
| 1 | | Not quite so much |
| 2 | | Only a little |
| 3 | | Hardly at all |
| | | |
| | | **I get a sort of frightened feeling as if something awful is about to happen:** |
| | 3 | Very definitely and quite badly |
| | 2 | Yes, but not too badly |
| | 1 | A little, but it doesn't worry me |
| | 0 | Not at all |
| | | |
| | | **I can laugh and see the funny side of things:** |
| 0 | | As much as I always could |
| 1 | | Not quite so much now |
| 2 | | Definitely not so much now |
| 3 | | Not at all |
| | | **Worrying thoughts go through my mind:** |
| | 3 | A great deal of the time |
| | 2 | A lot of the time |
| | 1 | From time to time, but not too often |
| | 0 | Only occasionally |
| | | |
| | | **I feel cheerful:** |
| 3 | | Not at all |
| 2 | | Not often |
| 1 | | Sometimes |
| 0 | | Most of the time |
| | | |
| | | **I can sit at ease and feel relaxed:** |
| | 0 | Definitely |
| | 1 | Usually |
| | 2 | Not Often |
| | 3 | Not at all |

| D | A | |
|---|---|---|
| | | **I feel as if I am slowed down:** |
| 3 | | Nearly all the time |
| 2 | | Very often |
| 1 | | Sometimes |
| 0 | | Not at all |
| | | |
| | | **I get a sort of frightened feeling like 'butterflies' in the stomach:** |
| | 0 | Not at all |
| | 1 | Occasionally |
| | 2 | Quite Often |
| | 3 | Very Often |
| | | |
| | | **I have lost interest in my appearance:** |
| 3 | | Definitely |
| 2 | | I don't take as much care as I should |
| 1 | | I may not take quite as much care |
| 0 | | I take just as much care as ever |
| | | |
| | | **I feel restless as I have to be on the move:** |
| | 3 | Very much indeed |
| | 2 | Quite a lot |
| | 1 | Not very much |
| | 0 | Not at all |
| | | **I look forward with enjoyment to things:** |
| 0 | | As much as I ever did |
| 1 | | Rather less than I used to |
| 2 | | Definitely less than I used to |
| 3 | | Hardly at all |
| | | |
| | | **I get sudden feelings of panic:** |
| | 3 | Very often indeed |
| | 2 | Quite often |
| | 1 | Not very often |
| | 0 | Not at all |
| | | |
| | | **I can enjoy a good book or radio or TV program:** |
| 0 | | Often |
| 1 | | Sometimes |
| 2 | | Not often |
| 3 | | Very seldom |

**FINALLY…**

Here are a selection of topics which people are often keen to discuss in the diabetes clinic. If you would like to talk about any of these issues, please circle them:

Using blood glucose monitor | Recording blood glucose results | Adjusting insulin | Injection sites

Carbohydrate counting | Correction doses | Repeat prescriptions | Getting appointments | Hypos

What to do when unwell | Diet | Exercise | Driving | Travel | Alcohol | Smoking | Feet

High blood sugars | HbA1c | Contraception | Eyes | Kidneys | Blood pressure | Cholesterol

**ESM questionnaire 2**

eced
Edinburgh Centre for Endocrinology & Diabetes

NHS Lothian

Freestyle Libre survey

**We would be grateful if you could answer all 30 questions. It shouldn't take much more than 7 minutes to complete the survey. We will use the information provided to help improve our diabetes service.**

1. Please enter your email address below (this will not be shared with anyone else):

2. Overall, how useful has the Libre been in helping control your diabetes?

0 (Not useful) 5 10 (Very useful)

3. Compared to finger-prick testing, how useful has the Libre been in helping adjust mealtime insulin doses?

0 (Not useful) 5 10 (Very useful)

4. Since you started using Libre has it changed the timing of your mealtime insulin doses?

- Earlier before meal - all meals
- Earlier before meal - some meals
- No change
- Later after meal - some meals
- Later after meal - all meals

5. Compared to finger-prick testing, how useful has the Libre been in helping adjust basal (background) insulin doses?

0 (Not useful) 5 10 (Very useful)

6. Compared to finger-prick testing, how useful has the Libre been in preventing DAY-TIME low blood glucose levels (i.e. less than 4)?

0 (Not useful) 5 10 (Very Useful)

7. Compared to finger-prick testing, how useful has the Libre been in preventing NIGHT-TIME low blood glucose levels (i.e. less than 4)?

0 (Not useful) 5 10 (Very useful)

8. Compared to finger-prick testing, how helpful has the Libre been in helping you TREAT low glucose (less than 4) levels?

0 (Not helpful) 5 10 (Very helpful)

9. Compared to finger-prick testing, how helpful has the Libre been in helping you exercise?

0 (Not helpful) 5 10 (Very helpful)

10. Since starting using Libre, what overall effect has it had on your diabetes control?

-5 (Less control) 0 (No change in control) 5 (More control)

11. **Compared to before you used Libre**, to what extent do you feel diabetes is taking up too much of your mental and physical energy each day?

- Much less of a problem now
- Less of a problem now
- No change
- More of a problem now
- Much more of a problem now

12. **Compared to before you used Libre**, to what extent do you feel your doctor doesn't know enough about diabetes and diabetes care?

- Much less of a problem now
- Less of a problem now
- No change
- More of a problem now
- Much more of a problem now

13. **Compared to before you used Libre**, to what extent do you feel angry, scared and/or depressed when you think about living with diabetes?

- Much less of a problem now
- Less of a problem now
- No change
- More of a problem now
- Much more of a problem now

14. **Compared to before you used Libre**, to what extent do you feel that your doctor doesn't give you clear enough directions on how to manage your diabetes?

- Much less of a problem now
- Less of a problem now
- No change
- More of a problem now
- Much more of a problem now

15. **Compared to before you used Libre**, to what extent do you feel that you are not testing your glucose levels frequently enough?

- Much less of a problem now
- Less of a problem now
- No change
- More of a problem now
- Much more of a problem now

16. **Compared to before you used Libre**, to what extent do you feel that you are often failing with your diabetes routine?

- Much less of a problem now
- Less of a problem now
- No change
- More of a problem now
- Much more of a problem now

17. **Compared to before you used Libre**, to what extent do you feel that that friends or family are not supportive enough of your self-care efforts?

- Much less of a problem now
- Less of a problem now
- No change
- More of a problem now
- Much more of a problem now

18. **Compared to before you used Libre**, to what extent do you feel that diabetes controls your life?

- Much less of a problem now
- Less of a problem now
- No change
- More of a problem now
- Much more of a problem now

19. **Compared to before you used Libre**, to what extent do you feel that your doctor doesn't take your concerns seriously enough?

- Much less of a problem now
- Less of a problem now
- No change
- More of a problem now
- Much more of a problem now

20. **Compared to before you used Libre**, to what extent do you **not** feel confident in your day-to-day ability to manage your diabetes?

- Much less of a problem now
- Less of a problem now
- No change
- More of a problem now
- Much more of a problem now

21. **Compared to before you used Libre**, to what extent do you feel that you will end up with serious long-term complications, no matter what you do?

- Much less of a problem now
- Less of a problem now
- No change
- More of a problem now
- Much more of a problem now

22. **Compared to before you used Libre**, to what extent do you feel that you are not sticking closely enough to a good meal plan?

- Much less of a problem now
- Less of a problem now
- No change
- More of a problem now
- Much more of a problem now

23. **Compared to before you used Libre**, to what extent do you feel that friends or family don't appreciate how difficult living with diabetes can be?

- Much less of a problem now
- Less of a problem now
- No change
- More of a problem now
- Much more of a problem now

24. **Compared to before you used Libre**, to what extent do you feel overwhelmed by the demands of living with diabetes?

- Much less of a problem now
- Less of a problem now
- No change
- More of a problem now
- Much more of a problem now

25. **Compared to before you used Libre**, to what extent do you feel you don't have a doctor who you can see regularly enough about your diabetes?

- ◯ Much less of a problem now
- ◯ Less of a problem now
- ◯ No change
- ◯ More of a problem now
- ◯ Much more of a problem now

26. **Compared to before you used Libre**, to what extent do you **not** feel motivated to keep up your diabetes self-management?

- ◯ Much less of a problem now
- ◯ Less of a problem now
- ◯ No change
- ◯ More of a problem now
- ◯ Much more of a problem now

27. **Compared to before you used Libre**, to what extent do you feel that friends or family don't give you the emotional support you would like?

- ◯ Much less of a problem now
- ◯ Less of a problem now
- ◯ No change
- ◯ More of a problem now
- ◯ Much more of a problem now

28. Regarding the **'Libre Start'** event you attended at the Royal Infirmary - how useful did you find this event:

0 (Not useful) 5 10 (Very useful)

29. Please leave any specific feedback you have regarding the **'Libre Start' event** (e.g. What was good? What could have been better?)

30. Please leave specific feedback you have about **your experience with the Freestyle Libre** (e.g. What impact has it had on your life? What is good about the system? What could be better?)
